# Effects of a Phytogenic Supplement Containing Olive By-Product and Green Tea Extracts on Growth Performance, Lipid Metabolism, and Hepatic Antioxidant Capacity in Largemouth Bass (*Micropterus salmoides*) Fed a High Soybean Meal Diet

**DOI:** 10.3390/antiox11122415

**Published:** 2022-12-07

**Authors:** Jiacheng Liu, Min Xue, Sofia Morais, Maolong He, Hao Wang, Jie Wang, Jose J. Pastor, Rui A. Gonçalves, Xiaofang Liang

**Affiliations:** 1 National Aquafeed Safety Assessment Center, Institute of Feed Research, Chinese Academy of Agricultural Sciences, Beijing 100081, China; 2 Animal Science Unit, Innovation Division, Lucta S.A., 08193 Bellaterra, Spain; 3 Innovation Division, Lucta (Guangzhou) Flavours Co., Ltd., Guangzhou 510530, China; 4 Feed Processing and Quality Control Innovation Team, Institute of Feed Research, Chinese Academy of Agricultural Sciences, Beijing 100081, China

**Keywords:** largemouth bass, soybean meal, phytogenic feed additive, antioxidant capacity, lipid metabolism

## Abstract

A 10-week growth trial was conducted to investigate the effects of a phytogenic feed additive (PFA) containing olive by-products and green tea extracts supplemented to a reduced fishmeal/high soybean meal diet on the growth performance, hepatic antioxidant capacity, lipid metabolism, and liver health of largemouth bass (*Micropterus salmoides*). Three experimental diets were tested: (1) a control high fishmeal (40%) and low soybean meal (15.57%) diet (named HFM), (2) a reduced fishmeal (30%) and high soybean meal (30.97%) diet (named HSB), and (3) a HSB diet supplemented with the PFA at 500 mg/kg (named HSB+P). Each diet was assigned to four replicate tanks, each containing 30 largemouth bass (initial body weight, IBW = 48.33 ± 0.01 g). The results showed that increasing the soybean meal content in the diet did not negatively affect growth performance, whereas supplementation with PFA significantly increased weight gain and specific growth rate of largemouth bass compared to both HFM and HSB groups. Reducing fishmeal and increasing soybean meal in the diet caused oxidative stress with a higher content of ROS in the liver. However, the hepatic antioxidant capacity was enhanced, with reduced ROS and increased GSH-Px levels in the HSB+P group. Moreover, the decrease of plasma TG, LDL-C, and LDL-C/TC, and downregulation of lipogenesis and cholesterol synthesis gene expression in liver, indicated that supplementation with the PFA improved fish lipid metabolism. Protein retention efficiency was also significantly increased in largemouth bass fed the diet with PFA supplementation, which regulated (enhanced) AKT-mTOR phosphorylation. These results clearly indicated that a PFA containing olive by-product and green tea extracts can positively improve growth performance, protein retention efficiency, antioxidant capacity, and lipid metabolism of largemouth bass fed a reduced fishmeal/high soybean meal diet.

## 1. Introduction

In 2019, aquaculture production was 85.3 million tons, an increase of 3.7 percent from 2018 [1]. With the rapid development of aquaculture and increase in world aquafeed production in the past decade, the inclusion levels of alternative protein sources have been rising to alleviate the shortage of fishmeal (FM), which was once the main protein source of aquafeeds but is now used much more judiciously [2]. Soybean meal (SBM) is the most commonly used alternative protein source in aquafeeds due to its relatively high protein content, favorable price, stable supply, balanced amino acid profile, and high digestibility [3]. Therefore, SBM has been commonly used as a major ingredient in many fish diets such as red snapper (*Lutjanus campechanus*) [4], red sea bream (*Pagrus major*) [5], rainbow trout (*Oncorhynchus mykiss*) [6], and largemouth bass (*Micropterus salmoides*) [7]. However, due to the presence of anti-nutritional factors, nutritional imbalances, and other shortcomings, excessive levels of SBM in feeds for aquatic animals often cause negative effects such as decreased feed intake and growth performance, oxidative stress, enteritis, and metabolic liver disease, especially in carnivorous fish [8,9,10].

Numerous studies have searched for new feed additives as an aid to alleviate oxidative stress and metabolic disease in fish, especially with the growing awareness of welfare in the aquaculture industry. Many phytogenic feed additives (PFAs) from natural plant extracts rich in polyphenols, triterpenoids, or flavonoids have various positive effects on cultured animals, including appetite stimulation, antimicrobial and antipathogenic action, immunostimulation, growth promotion, and antioxidant action [11,12,13,14,15]. Olive (*Olea europaea*) is one of the oldest woody tree species widely cultivated in the Mediterranean area [16]. Green tea (*Camellia sinensis*) is one of the most popular drinks around the world and is planted mainly in east Asia [17]. Recent studies have shown multiple beneficial effects of additives extracted from olive or green tea on fish growth and metabolism in aquaculture. Dietary supplementation with olive leaf extract, in which the main active components are polyphenols, enhanced the growth performance of common carp (*Cyprinus carpio*) by activating digestive enzyme activity in the intestine and growth-related gene expression [18]. Dietary supplementation of polyphenols extracted from chestnut and olive positively affected the innate immune response, oxidative status, and growth performance of common carp [19]. Dietary olive leaf extract improved immunity and disease resistance to *Yersinia ruckeri* infection in rainbow trout [20]. Decaffeinated green tea (*Camellia sinensis*) also enhanced the immunity of rainbow trout against *Yersinia ruckeri* [21]. Moreover, a triterpenoid-rich olive extract (OE) has recently been reported to improve the antioxidant and anti-inflammatory capacity, and positively modulate lipid metabolism, to relieve the oxidative stress and lipid metabolic disorder of largemouth bass fed high-starch feed [22].

Largemouth bass (*Micropterus salmoides*) is a carnivorous fish native to North America that was introduced in China in 1983. Because of its fast growth and high commercial value, largemouth bass is reared in large quantities in freshwater culture systems [23]. In recent years, the elevated incidence of metabolic disease in largemouth bass caused by the replacement of high levels of FM with plant proteins in commercial feeds has hindered production growth and fish welfare. Research showed that SBM could replace 30% FM (with 24.5% FM and 24.46% SBM in diet) without negative impacts on growth performance and protein retention in largemouth bass [7], but higher reductions of FM through SBM inclusion should be avoided. Many studies focused on testing different functional feed additives such as bile acids, yeast hydrolysates, synbiotics, or butylated hydroxytoluene, aiming to solve this problem which limits the development of the industry [24,25,26,27]. Available evidence suggests that triterpenes and polyphenols from phytogenic extracts have good potential to be used as functional feed additives in aquafeeds. Therefore, the purpose of our study was to evaluate the effects of a PFA obtained from olive by-product and green tea extracts on growth performance, oxidative stress, hepatic health, and lipid metabolism of largemouth bass fed with a reduced FM and high SBM diet.

## 2. Materials and Methods

### 2.1. Experimental Diets

Three isonitrogenous and isoenergetic experimental diets were formulated: (1) a high FM diet containing 40% FM and 15.57% SBM (named HFM) was used as the control group; (2) a lower FM and higher SBM diet containing 30% FM and 30.97% SBM (named HSB); and (3) a HSB diet supplemented with a PFA from Lucta S.A. (Barcelona, Spain) containing a standardized olive extract (>75% triterpenes by HPLC-UV) obtained from the by-products of the olive table industry by a patented methodology (WO20100072874A1) and a green tea extract (98% polyphenols) in a ratio of 2.3 to 1, dosed at 500 mg/kg (named as HSB+P). The feed ingredients were ground into fine powder through a 247 μm mesh. Each diet was processed into 2 mm-diameter floating pellets under the following extrusion condition: feeding section (90 °C/5 s), compression section (130 °C/5 s), and metering section (150 °C/4 s), using a twin-screwed extruder (EXT50A, YangGong Machine, Beijing, China). The formulation and composition of experimental diets is shown in Table 1.

### 2.2. Fish and Rearing Conditions

Largemouth bass were obtained from the National Fisheries Technology Extension Center (Beijing, China). A 70-day feeding trial was conducted in the indoor circulating water system at the National Aquafeed Safety Assessment Center of IFR, CAAS (Beijing, China). All the fish were acclimated to the system for 14 days and fed a commercial diet before the formal feeding trial. Fish (initial body weight 48.33 ± 0.01 g) were randomly selected and transferred to 12 tanks (256 L) with 30 fish per tank and 4 tanks per treatment. During the experiment, fish were fed to apparent satiation twice a day (8:00 a.m. and 4:00 p.m.). Feed intake was recorded daily. Water temperature was measured twice a day and water quality parameters were measured every 3 days. The tanks in the system had aeration to maintain dissolved oxygen. Water parameters were recorded as follows: water temperature, 25–27 °C; ammonia-N < 0.4 mg L^−1^; NO_2_^−^ < 0.3 mg L^−1^; dissolved oxygen above 5 mg L^−1^; pH around 7.0.

### 2.3. Sampling

Before the start of the experiment, an initial sample of three fish was taken and kept in the freezer at −20 °C. At the end of the experiment, all fish were starved for 24 h. Three fish per tank were then collected and kept at −20 °C to analyze the final whole-body composition. Sixteen fish per treatment (four fish per tank) were anesthetized with chlorobutanol (300 mg/mL) for sampling, including measurements of body length, body weight, viscera, and liver weight. The remaining fish of each tank were counted and batch-weighted to analyze growth performance. Blood samples were drawn from the tail vein of the fish using 2% sodium fluoride (SINOPHARM, Beijing, China) and 4% potassium oxalate (SINOPHARM, Beijing, China) as anticoagulation agents, and then immediately centrifuged at 4 °C (4000 rpm, 10 min). Plasma samples were kept in the −40 °C freezer. Two liver samples were collected near the bile duct. One was put into a 1.5 mL RNase-free tube (Axygen, Waltham CA, USA), immediately frozen in liquid nitrogen, and kept in a −80 °C freezer pending mRNA extraction and enzymatic analysis. The other (0.5 mm length) was fixed with 4% paraformaldehyde solution for histological examination.

### 2.4. Chemical Analysis

Dry matter was measured by drying the samples at 105 °C to a constant weight. Crude protein (N*6.25) was measured by the Kjeldahl method, using a K1100F automatic Kjeldahl analyzer (Hanon, Jinan, China) to measure N content after acid digestion of the samples with a SH420 Kjeldahl digestor (Hanon, Jinan, China). Crude lipid was measured by the method of ether extraction using the Soxtex System 1043 (Foss, Hillerød, Denmark). Ash was measured by putting the samples in a muffle furnace at 550 °C for 4 h. Gross energy was determined using an IKAC2000 Calorimeter (IKA, Staufen, Germany).

### 2.5. Hematological and Hepatic Biochemical Parameters Analysis

Plasma or hepatic glucose (Glu), total cholesterol (TC), triglyceride (TG), high-density lipoprotein cholesterol (HDL-C), low-density lipoprotein cholesterol (LDL-C), total bile acid (TBA), total protein (TP), alanine aminotransferase (ALT), aspartate aminotransferase (AST), and alkaline phosphatase (AKP) were determined by assay kits (Nanjing Jiancheng Co., Nanjing, China), according to the supplier’s instructions.

### 2.6. Hepatic Antioxidative Parameters Analysis

Liver samples were analyzed for total antioxidant capacity (T-AOC), total superoxide dismutase (T-SOD), catalase (CAT), malondialdehyde (MDA), and glutathione peroxidase (GSH-Px) activities using assay kits (Nanjing Jiancheng Co., Nanjing, China) according to the supplier’s instructions. The level of reactive oxygen species (ROS) was measured by an ELISA kit (Jiangsu Meimian Industrial Co., Ltd.; Yancheng, China) following the supplier’s instructions.

### 2.7. RNA Isolation, Reverse Transcription, and Real-Time PCR (RT-qPCR)

Total RNA from liver was isolated using RNAiso Plus reagent (No. D9108A, Takara, Tokyo, Japan). Determination of total RNA was performed by spectrophotometry using a NanoDrop 2000 (Thermo, Waltham, CA, USA). Then, electrophoresis on a 1% denaturing agarose gel was used to evaluate the integrity of the RNA. For each reverse transcription reaction, 1.0 μg of total RNA was first treated with gDNA Eraser to remove genomic DNA contaminants and then subjected to cDNA synthesis by reverse transcription in a 20 μL volume using the manuscript cDNA Synthesis Kit (Bio-Rad, Hercules, CA, USA).

The gene sequences of largemouth bass were obtained from an RNA-seq database. EF1α (GenBank accession no. JQ995147) is a housekeeping gene whose expression was not affected by treatment in this experiment and was used as an endogenous reference to standardize the amount of template. Specific primers of genes used for mRNA quantification by RT-qPCR are shown in Table 2. The RT-qPCR was conducted in a CFX96TM Real-time System (Bio-Rad, Hercules CA, USA). The reaction volume was 20 μL and contained iTaqTM Universal SYBR^®^ Green Supermix (Bio-Rad, Hercules CA, USA).

Serial dilutions of cDNA generated from liver tissues were used to draw a standard curve to determine the amplification efficiency (E-value) of reference and target genes. The E-value ranged from 90% to 110% (Table 2). The RT-qPCR temperature profile for all genes was 95 °C for 30 s, followed by 40 cycles of 10 s at 95 °C, 30 s at Tm (Table 2), and 40 s at 72 °C. After the final cycle of PCR, the melting curves were systematically monitored (65 °C temperature gradient at 0.05 °C/10 s from 65 °C to 95 °C). Each sample was run in triplicate. PCR-grade water in place of the template served as the negative control. The RT-qPCR data were analyzed using the 2^−ΔΔCt^ method [28]. The mRNA levels of target genes were shown as the n-fold difference compared to the calibrator.

### 2.8. Western Blot

Liver tissue was extracted in RIPA Reagent (Beyotime, Shanghai, China) with phosphatase and protease inhibitor cocktail (Cell Signaling Technology, Boston, MA, USA). The density of protein was measured by a BCA Protein Quantification Kit (Bio-Rad, CA, USA). Automated western blots were performed by a Jess^TM^ capillary western blot system (ProteinSimple, Santa Clara, CA, USA) using pre-filled plates (12–230 kDa and 44–660 kDa) according to the manufacturer’s instructions. The primary antibodies were β-tubulin (Cell Signaling Technology, #2146, 1:50 dilution), AKT (Cell Signaling Technology, #9272, 1:50 dilution), P-AKT (Ser 473) (Cell Signaling Technology, #4060, 1:300 dilution), mTOR (Cell Signaling Technology, #2972, 1:100 dilution), and P-mTOR (Ser 2448) (Cell Signaling Technology, #2971, 1:50 dilution).

### 2.9. Hepatic Histopathological Examination

The liver samples were fixed for 24 h, and then dehydrated, embedded in paraffin, cut to a thickness of 6 μm, and stained by hematoxylin-eosin (H&E). All the images were automatically identified and captured using a TissueFAXS System (TissueGnostics, Vienna, Austria), and then observed to assess the presence of fatty liver or fibrosis phenotypes.

### 2.10. Statical Analysis

Differences among the three groups were analyzed by one-way ANOVA using SPSS statistics 22.0 (SPSS Inc., New York, NY, USA). Normality and homogeneity of variance were confirmed by Shapiro–Wilk and Levene’s test before ANOVA, respectively. If the homogeneity of variance test failed, data were transformed, or the Brown–Forsythe and Welch ANOVA tests were used instead. Differences among the means were analyzed by Duncan’s multiple range test and considered significant when *p* < 0.05. All data were presented as mean value ± standard error of the mean (S.E.M). Graphs were prepared using GraphPad Prism 8.3.0 (GraphPad Software Inc., San Diego, CA, USA).

## 3. Results

### 3.1. Growth Performance, Morphometric Parameters, and Whole-Body Proximate Composition

The survival rate (SR) of largemouth bass was 100% in all groups. Final body weight (FBW), specific growth rate (SGR), and weight gain rate (WGR) of the fish in the HSB+P group were significantly higher than those in the HFM and HSB groups (*p* < 0.05), while there were no significant differences between the HFM and HSB groups (*p* > 0.05). The FCR was highest in the HSB group (1.31 ± 0.05) and lowest in the HSB+P group (1.22 ± 0.02), although the difference was not statistically significant (*p* > 0.05). The different experimental diets had no effect on feeding rate (FR) (*p* > 0.05). Protein retention efficiency (PRE) was highest in the HSB+P group, significantly compared to the HSB group (*p* < 0.05), but not significantly different from the HFM group. The morphometric parameters and whole-body nutrient compositions of largemouth bass did not show significant differences among the three groups (Table 3 and Table 4; *p* > 0.05).

### 3.2. Plasma Biochemical Parameters

The level of Glu in the HSB group was significantly lower than in the HSB+P group (Table 5, *p* < 0.05). Compared with the HFM group, reduced FM and higher SBM levels in the diet significantly increased plasma TG, LDL-C, and LDL-C/TC (*p* < 0.05). These three indicators were significantly decreased after the addition of the PFA (*p* < 0.05), showing no differences to the HFM group, except in LDL-C content, which was significantly lower in HSB+P than in the HFM group (*p* < 0.05). The plasma level of HDL-C in the HFM group was significantly higher than in the HSB group but was comparable to the HSB+P group. The plasma levels of TC, HDL-C/TC, TBA, and TP showed no significant differences among the three groups (*p* > 0.05).

### 3.3. Liver Function and Hepatic Antioxidant Capacity

Parameters related to the liver function of largemouth bass are shown in Table 6. The TBA content in the HFM and HSB+P groups was significantly lower than in the HSB group (*p* < 0.05). Liver AST, ALT, AKP, and TP did not show marked differences among the three groups (*p* > 0.05).

Hepatic antioxidant capacity parameters are shown in Table 7. Liver ROS, T-AOC, and CAT were significantly increased in the HSB group compared with the other two treatments (*p* < 0.05). Significant differences were observed between the three groups in GSH-Px, with the HSB+P group having the highest activity, followed by the HFM group, and HSB group showed the lowest activity (*p* < 0.05). There were no significant differences in hepatic SOD and MDA among the three groups (*p* > 0.05).

### 3.4. Hepatic Lipid Metabolism

**Figure 1:**
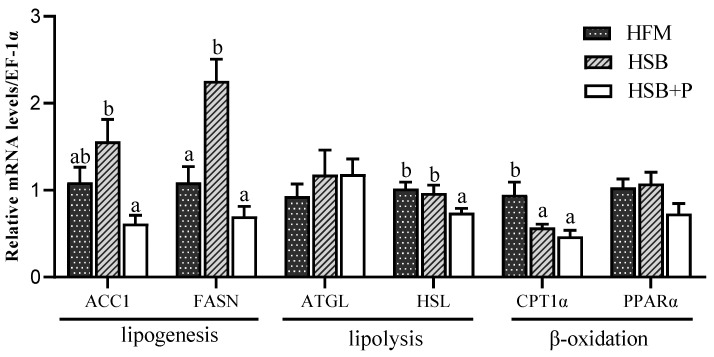
Relative mRNA levels of lipid metabolism genes in liver tissue of largemouth bass. The lipogenesis genes ACC1 and FASN, lipolysis gene HSL, and β-oxidation gene CPT1α were downregulated in the HSB+P group (Mean ± SEM, *n* = 8). ^ab^ Values with no or the same superscript letter mean no significant differences (*p* > 0.05), while different superscript letters indicate significant differences (*p* < 0.05).

The mRNA expression of the lipogenesis gene FASN was significantly downregulated in the HSB+P and HFM groups compared to the HSB group (Figure 1, *p* < 0.05). The mRNA level of ACC1 was also significantly downregulated in the HSB+P group compared with the HSB group, while HFM presented intermediate levels and was not significantly different from the other groups. The mRNA level of the lipolysis gene HSL was significantly downregulated in HSB+P compared to the HSB and HFM groups (*p* < 0.05). On the other hand, the β-oxidation gene CPT1α was significantly upregulated in the HFM group compared to the other two groups (*p* < 0.05).

### 3.5. Hepatic Histopathological Examination

**Figure 2:**
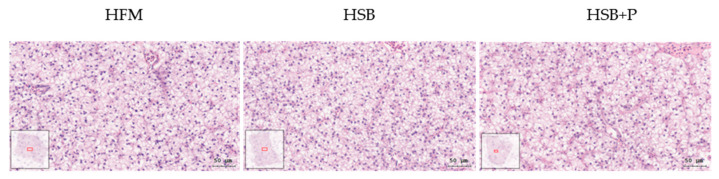
Histopathological examination under H&E (hematoxylin and eosin) staining of largemouth bass liver samples (scale bar: 50 μm, *n* = 12). The hepatocytes presented a normal shape and did not indicate fatty liver or other anomalies.

Liver tissue samples of largemouth bass were examined under a 400× visual field after H&E staining (Figure 2). Twelve samples were observed for each group and all samples showed a normal phenotype. The hepatocytes of the tissue were all in good shape and did not show fatty liver phenotype or enlarged and vacuolated cells.

### 3.6. Western Blot for AKT-mTOR Pathway

**Figure 3:**
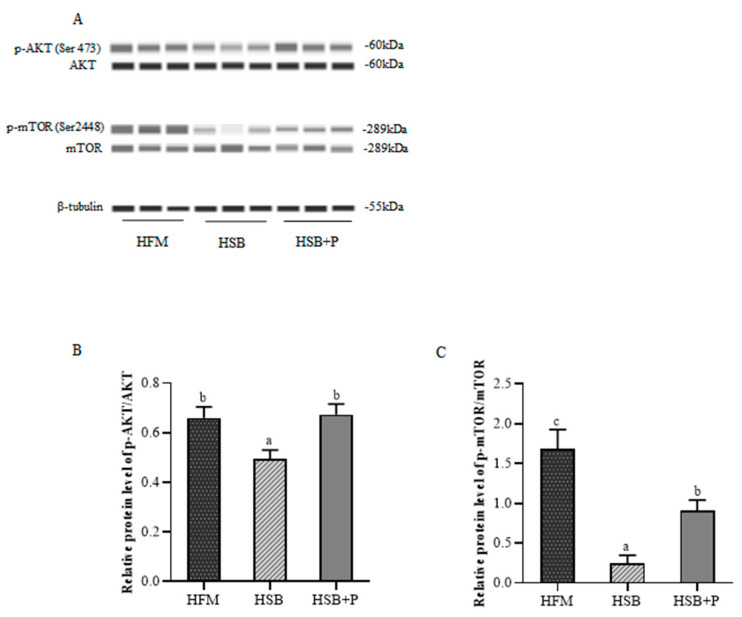
Western blot results of total and phosphorylated AKT and mTOR (Mean ± SEM, *n* = 3). (**A**) The addition of the phytogenic mixture promoted AKT and mTOR protein phosphorylation in liver of largemouth bass; (**B**) Relative protein levels of p-AKT/AKT; (**C**) Relative protein levels of p-mTOR/mTOR. The protein expression of phosphorylated mTOR and AKT were significantly increased after the addition of the phytogenic supplement. ^abc^ Values with no or the same superscript letter mean no significant differences (*p* > 0.05), while different superscript letters indicate significant differences (*p* < 0.05).

Protein expression levels of mTOR signaling pathways were measured in the largemouth bass liver and results are shown in Figure 3. Compared to the HFM group, reducing FM and increasing SBM contents in the diet significantly downregulated the phosphorylation of AKT and mTOR. However, protein expression ratios of p-AKT to AKT and p-mTOR to mTOR were significantly increased after the addition of the phytogenic supplement (*p* < 0.05).

## 4. Discussion

Due to the need to produce rising volumes of feeds with lowering availability and increasing price of FM, SBM is the most commonly used protein alternative in the diets of most farmed fish. Our results showed that replacing 25% of FM (FM was reduced from 40% to 30%) while raising the level of SBM from 15.57% to 30.97% did not negatively affect the growth performance of largemouth bass. This was in line with the results of some previous studies. There were no significant differences in WGR and SGR of obscure puffer (*Takifugu obscurus*) when SBM was used to replace FM at levels below 30%, in which FM was reduced from 50% to 35% and SBM was increased from 0 to 20% [29]. Another study showed that FM could be reduced from 60% to 48% by 14.4% fermented soybean meal protein (FSBM) replacement in black sea bream (*Acanthopagrus schlegelii*) diets. However, when the substitution level increased to 30% (diet containing 42% FM and 21.6% FSBM), the apparent digestibility of nutrients, WGR and SGR, were significantly decreased [30]. Such results indicate that dietary SBM inclusion does not affect the growth performance of fish up to a certain level, but a high proportion of replacement of FM by SBM can lead to some adverse effects, due to the natural presence of various antinutritional factors in SBM. A high substitution of FM by SBM (usually ≥ 75%) has been shown to induce severe negative effects on feed intake, growth, activity of digestive enzymes, feed utilization, and liver and intestinal health in fish, such as Japanese seabass (*Lateolabrax japonicus*) and tilapia (*Oreochromis niloticus*) [8,31]. Similarly, results from this study showed that even if there was no significant difference in growth performance under controlled laboratory conditions, the reduction from 40% to 30% of FM and increase from 15.57% to 30.97% of SBM in largemouth bass diet caused some negative effects, including a tendency for decreased PRE and worsened FCR, and significant changes in plasma biochemical parameters, in at least one liver function parameter (TBA), in oxidative stress parameters and in lipid metabolism. Therefore, it is plausible that under more challenging and stressful culture conditions, these physiological changes could result in lower fish performance.

Previous studies testing plant extracts as dietary supplements, including olive and green tea extracts, showed contradictory effects on growth performance in fish. For instance, an olive leaf extract did not have an obvious effect on the growth performance of rainbow trout and common carp [20,32]. Green tea administration also showed no obvious effect on growth of rainbow trout [33]. Nevertheless, other studies indicated that dietary supplementation with olive waste cake and olive leaf extract can have a positive effect on the growth performance of rainbow trout and common carp [34,35]. Interestingly, our experiment found that the supplementation of largemouth bass diet with 0.05% of a PFA containing plant extracts derived from olive and green tea, having main active components triterpenes (maslinic acid and oleanolic acid) and polyphenols, can not only significantly improve growth performance of largemouth bass but can also regulate mammalian target of rapamycin (mTOR) and AKT (serine/threonine kinase, also known as protein kinase B) to promote protein deposition. The mTOR is a crucial regulator of cell growth, survival, and metabolism, and AKT can directly phosphorylate (activate) mTOR at Ser 2448 [36,37]. A large body of evidence indicates that an AKT/mTOR signaling deficiency is frequently associated with defects in protein synthesis control [38]. The replacement of FM with SBM or mixed plant protein could lead to hypoactive TOR signaling on turbot (*Scophthalmus maximus* L.) [39,40]. Our experiment also confirmed that the ratios of phosphorylated (activated) mTOR to mTOR, and of phosphorylated (activated) AKT to AKT, were decreased in largemouth bass after the high SBM inclusion in the HSB group. The addition of the phytogenic supplement significantly alleviated this situation, as both p-AKT/AKT and p-mTOR/mTOR were increased in the HSB+P compared to the HSB group. This may lead to higher cell proliferation and differentiation and thereby explain, at least partly, the enhanced PRE and growth of largemouth bass fed the HSB+P diet.

Bile acids (BAs) have an important function in facilitating the absorption of lipid nutrients and can also degrade toxins and endotoxins, protecting the fish liver from damage associated with high inclusion of plant proteins in fish diets, especially in carnivorous and omnivorous species [41,42]. Bile acid metabolism has been shown to be generally modified when fish are fed on plant ingredients [43]. However, excessive synthesis of bile acids in the liver can lead to these reaching toxic levels, causing cholestasis and hepatic damage [44]. In the present research, the significant and very marked rise in TBA in the liver of largemouth bass fed the HSB diet was associated with a risk of hepatic cholestasis, accompanied by hyperlipidemia and oxidative damage, although the results of the hepatic histopathological examination were normal. The supplementation of this diet with 0.05% of PFA was able to significantly decrease the TBA in liver up to the HFM treatment level.

Concurrently, abnormal bile acid metabolism is often accompanied by lipid metabolism disorders, such as excessive lipid accumulation affecting the nutritional composition, atherogenic lipid profile and health of the fish [44,45,46]. Many previous studies also indicated that diets containing high SBM levels increased the risk of lipid metabolism disorders in fish and imbalances were often manifested in an increase in plasma TC and TG. With increasing levels of dietary SBM, the concentration of TC, TG, and LDL-C in plasma of Japanese flounder (*Paralichthys olivaceus*) increased, while HDL-C concentration decreased [47]. Moreover, plasma TG increased when spotted rose snapper (*Lutjanus guttatusalso*) were fed a 60% SBM diet [48]. In our experiment, the elevated plasma concentrations of TG and LDL-C, and of the LDL-C/TC ratio in the HSB group, suggested a lipid metabolism disorder in largemouth bass fed the HSB diet compared to the HFM and HSB+P groups. Additionally, substitution of FM with SBM in this study resulted in significant changes in the expression of important lipid metabolism genes, such as the downregulation of hepatic lipid β-oxidation gene CPT1α and upregulation of liver lipogenesis genes FAS and ACC1 (although only significantly different from the HSB+P group), which are key enzymes involved in de novo lipogenesis [49]. By supplementing the HSB diet with the PFA, altered plasma biochemical parameters, including the concentration of TG and LDL-C, and LDL-C/TC ratio, were decreased to levels similar to the HFM group. A similar reduction in plasma TG and TC was observed in Nile tilapia fed diets supplemented with increasing concentrations of an olive extract [50]. The phytogenic supplement functioned mainly by downregulating liver lipogenesis genes ACC1 and FASN and reducing plasma TG concentration in largemouth bass. ATGL is a key enzyme in the first step of TG catabolism, followed by TG degradation into glycerol and fatty acids under the action of HSL [51,52]. The downregulation of liver HSL was associated to a reduced fatty acid synthesis in the HSB+P group. Therefore, we can conclude that lipid synthesis and lipolysis were in a balanced state. On the other hand, CPT1α is involved in lipid β-oxidation. The downregulation of CPT1α in the HSB group could be responsible for the lipid accumulation in this group. However, CPT1α was also downregulated after the addition of PFA, which in this case was most likely caused by the reduction in fatty acid synthesis. These results are supported by our previous study in largemouth bass which showed that a similar triterpene-rich olive extract can regulate lipid metabolism by inhibiting the expression of liver lipogenesis genes ACC1 and FASN [22]. Meanwhile, as a feed additive, tea polyphenols have been shown to regulate lipid deposition in tilapia, to promote glucose utilization in grass carp (*Ctenopharyngodon idella*) and to reduce serum TC and TG in large yellow croaker (*Larimichthys crocea*) [53,54,55]. Another previous experiment reported that a green tea extract decreased serum LDL-C in olive flounder (*Paralichthys olivaceus*) [56]. Furthermore, the addition of a green tea ethanol extract to juvenile black rockfish (*Sebastes schlegeli*) feed was reported to reduce serum TC in a dose-dependent manner [57]. Similar effects have also been reported in mammals. For instance, a study with FL83B mouse hepatocytes reported that Chinese olive fruits have the potential to improve metabolic abnormalities associated with fatty liver by suppressing lipogenesis genes ACC1, FAS, and SREBP1-c [58]. Finally, researchers believe that the lowest mortality rates from cardiovascular diseases observed in the Mediterranean area are at least partly explained by the consumption of high levels of olives and olive oil, which can increase high-density lipoprotein cholesterol concentrations to reduce cardiovascular disease risk [59]. The high catechins in green tea can decrease body fat and LDL-C, therefore also contributing to a reduction of cardiovascular and obesity risk in humans [60,61].

The presence of anti-nutritional factors in SBM and the hepatic lipid accumulation that it frequently induces, when included in fish diets at high levels, is often a cause of oxidative stress in fish [62]. For instance, when FM was reduced from 20% to 10% and SBM was increased from 0% to 15% in tilapia diets, the activity of superoxide dismutase (SOD) in tilapia was decreased [31]. In this study, the important rise in SBM level, concomitant with the 25% reduction in FM, caused a higher hepatic oxidative stress in largemouth bass, which was reflected in an increase in ROS in the HSB group. Triterpenes and polyphenols such as coumaric, ferulic, cinnamic acid, and oleuropein, to mention just a few, are active and effective molecules to reduce oxidative stress, inflammatory responses and can also act as radical scavengers [63,64,65]. A previous study reported that adding 5 g/kg of dietary polyphenols from chestnut wood extract and olive extract to the feed of Asian sea bass (*Lates calcarifer*) could enhance liver antioxidant enzyme activity and reduce MDA level [66]. Dietary supplementation with an olive extract was also previously shown to reduce liver MDA concentration in largemouth bass [22]. Green tea extracts, on the other hand, were shown to have protective effects from the adverse consequences of oxidized oil in a sturgeon hybrid of Sterlet (*Huso huso* ♂ × *Acipenser ruthenus* ♀) [67]. In addition, oxidative stress can usually be relieved with the enhancement of lipid metabolism in largemouth bass [24,27]. Hence, it was not surprising to find that the addition of 500 mg/kg of the PFA to the HSB diet in this study was able to alleviate hepatic oxidative stress, indicated by the reduced ROS levels and enhanced liver GSH-Px concentration, as well as by the improvement of lipid metabolism in largemouth bass.

## 5. Conclusions

Our experiment indicated that when FM level was reduced by 25% (from 40% to 30%) and replaced by SBM (practically doubling, from 15.57% to 30.97%) in largemouth bass diets, this caused oxidative stress and lipid metabolism burden to the fish, even though growth performance and hepatic histopathological examination did not show obvious differences to the HFM control. However, these negative effects could be completely reverted by dietary supplementation with 500 mg/kg of a PFA containing an olive by-product and green tea extracts. Furthermore, the supplementation considerably improved growth performance, even significantly above the HFM positive control, and enhanced protein retention efficiency.

## Figures and Tables

**Table 1:** Formulation and compositions of experiment diets (g/kg).

Ingredients	HFM	HSB	HSB+P
Fishmeal ^a^	400	300	300
Soybean meal ^b^	155.7	309.7	309.7
Cottonseed protein concentrate	50	50	50
Corn gluten meal	80	80	80
Enzymolysis fishmeal soluble	30	30	30
Wheat flour	76.6	77.6	76.6
Cassava starch	50	50	50
Fish oil	19.2	24.2	24.2
Soybean oil	60	60	60
Premix ^c^	14	14	14
Microcrystalline cellulose	60	0	0.5
Choline chloride	2	2	2
L-Lysine HCL	2	2	2
L-Taurine	0.5	0.5	0.5
Phytogenic supplement ^d^	0	0	0.5
Total	1000	1000	1000
Analyzed chemical composition (%, in as is basis)			
Crude protein (DM)	50.24	51.56	50.72
Crude lipid (DM)	10.89	10.15	10.52
Ash	8.50	8.33	7.90
Gross energy (MJ/kg)	20.96	21.29	21.79

^a^ Fish meal: steam dried, prime. Tecnológica de Alimentos S.A., Ltd. (Lima, Peru). ^b^ Soybean meal was purchased from Qingdao Bohai Biotechnology Co., Ltd. (Qingdao, China), which concluded 7S-conglycinin 45.55 mg/g and 11S-Glycinin 26.04 mg/g. ^c^ Premix (g.kg^−1^ diet): Vitamin Premix 5, Mineral Premix 2.5, Rovimix-stay-C, 3.5, Mold inhibitor 1, Mycotoxin binder 2. Vitamin Premix (per kg diet): Vitamin A, 6000IU; Vitamin D3, 1000IU; Vitamin E, 100IU; Biotin, 0.2 mg; Folic acid, 9 mg; Niacin, 200 mg; Pantothenate, 100 mg; Pyridoxine (B6), 25 mg; Riboflavin (B2), 40 mg; Thiamin (B1), 40 mg; Vitamin B12, 10 mcg; Ethoxyquin, 2.5 mg. Mineral Premix (per kg diet): Iron, 100 mg; Manganese, 25 mg; Copper, 10 mg; Zinc, 100 mg; Iodine, 4.5 mg; Cobalt, 0.05 mg; Selenium, 0.5 mg. ^d^ Phytogenic supplement: a mixture supplied by Lucta S.A. (Barcelona, Spain) containing a standardized olive extract (>75% triterpenes by HPLC-UV) obtained from the byproducts of the olive industry by a patented methodology (WO20100072874A1) and a green tea extract (98% polyphenols) in a ratio of 2.3 to 1.

**Table 2:** Primer sequences used for real-time quantitative PCR.

Gene	Primers	Sequence5′-3′	Target Size (bp)	E-Value (%)	TM (°C)
ACC1	F *	ATCCCTCTTTGCCACTGTTG	121	102.2	57.5
	R *	GAGGTGATGTTGCTCGCATA			
ATGL	F	CCATGATGCTCCCCTACACT	176	99.1	58.0
	R	GGCAGATACACTTCGGGAAA			
CPT1α	F	CATGGAAAGCCAGCCTTTAG	128	98.8	60.0
	R	GAGCACCAGACACGCTAACA			
EF1α	F	TGCTGCTGGTGTTGGTGAGTT	147	102.8	60.4
	R	TTCTGGCTGTAAGGGGGCTC			
FASN	F	TGTGGTGCTGAACTCTCTGG	121	102.1	57.5
	R	CATGCCTAGTGGGGAGTTGT			
HSL	F	ATCAGAGCTGGAGCACCCTA	122	99.3	60.0
	R	GCAGAGGAGAGCAGAAAGGA			
PPARα	F	CCACCGCAATGGTCGATATG	144	104.3	59.0
	R	TGCTGTTGATGGACTGGGAAA			

ACC1, acetyl-CoA carboxylase 1; ATGL, adipose triglyceride lipase; CPT1α, carnitine palmitoyltransferase 1α; EF1α, eukaryotic translation elongation factor 1α; FASN, fatty acid synthase; HSL, hormone-sensitive triglyceride lipase; PPARα, peroxisome proliferator activated receptor α. * F: forward primer; R: reverse primer.

**Table 3:** Effects of experimental diets on growth performance and morphometric parameters of largemouth bass (Mean ± SEM, *n* = 4).

	HFM	HSB	HSB+P
Growth performance ^#^			
IBW (g)	48.34 ± 0.01		
FBW (g)	124.18 ± 5.31 ^a^	122.34 ± 3.82 ^a^	142.29± 5.95 ^b^
SR (%)	100 ± 0.00	100 ± 0.00	100 ± 0.00
SGR (%/d)	1.34 ± 0.06 ^a^	1.31 ± 0.04 ^a^	1.55 ± 0.06 ^b^
WGR (%)	159.61 ± 11.01 ^a^	155.09 ± 7.89 ^a^	198.85 ± 12.30 ^b^
FCR	1.27 ± 0.01	1.31 ± 0.05	1.22 ± 0.02
FR (%bw/d)	1.37 ± 0.04	1.38 ± 0.01	1.38 ± 0.03
PRE (%)	27.86 ± 0.93 ^ab^	25.23 ± 1.09 ^a^	29.02 ± 0.49 ^b^
Morphometric parameters *			
CF	1.69 ± 0.02	1.75 ± 0.02	1.71 ± 0.04
HSI (%)	1.74 ± 0.28	1.61 ± 0.58	1.59 ± 0.34
VSI (%)	8.53 ± 0.25	8.27 ± 0.23	8.05 ± 0.22
VAI (%)	3.63 ± 0.19	3.25 ± 0.11	3.16 ± 0.18

^ab^ Values with no or the same superscript letter mean no significant differences in the same row (*p* > 0.05), while different superscript letters indicate significant differences (*p* < 0.05). ^#^ IBW: initial body weight. FBW: final body weight, *n* = 17. SR (Survival Rate, %) = 100 × final fish number/initial fish number, *n* = 4. SGR (Specific growth rate, %/d) = 100 × [Ln (FBW) − Ln (IBW)]/days, *n* = 17. WGR (Weight gain rate, %) = 100 × ((Final body weight − initial body weight + dead fish weight)/initial body weight), *n* = 17. FCR (Feed conversion ratio) = feed intake/(Wf + Wd − Wi). Wf is the final total weight, Wd is the total weight of dead fish, Wi is the initial total weight, *n* = 4. FR (Feeding rate, % body weight/d) = 100 × feed intake/((Wf + Wi + Wd)/2)/days, *n* = 4. PRE (Protein retention efficiency, %) = 100 × (final total weight × final fish protein content − initial total weight × initial fish protein content)/(total feed intake × feed protein content), *n* = 4. * CF (Condition factor) = 100 × (live weight, g)/(fork length3,cm), *n* = 16. HSI (Hepatosomatic index, %) = 100 × (liver weight)/body weight, *n* = 16. VSI (Viscerosomatic index, %) = 100 × (viscera weight)/body weight, *n* = 16. VAI (Visceral adipose index, %) = 100 × visceral adipose weight/whole body weight, *n* = 16.

**Table 4:** Effects of experimental diets on whole body proximate composition of largemouth bass (Mean ± SEM, *n* = 4).

	HFM	HSB	HSB+P
Moisture (%)	68.36 ± 0.49	68.80 ± 0.56	68.40 ± 0.57
Crude protein (%)	17.03 ± 0.26	16.55 ± 0.28	17.03 ± 0.07
Crude lipid (%)	8.51 ± 0.21	8.21 ± 0.27	8.03 ± 0.31
Ash (%)	3.77 ± 0.17	3.98 ± 0.12	3.91 ± 0.10
Gross Energy (MJ/kg)	8.22 ± 0.18	8.02 ± 0.19	8.11 ± 0.28

**Table 5:** Effects of experimental diets on plasma biochemical parameters of largemouth bass (Mean ± SEM, *n* = 8).

	HFM	HSB	HSB+P
Glu (mmol/L)	4.90 ± 0.35 ^ab^	4.66 ± 0.31 ^a^	5.93 ± 0.44 ^b^
TC (mmol/L)	8.79 ± 0.33	8.37 ± 0.81	7.06 ± 0.47
TG (mmol/L)	6.69 ± 0.52 ^a^	10.11 ± 1.05 ^b^	6.35 ± 0.59 ^a^
LDL-C (mmol/L)	0.99 ± 0.04 ^b^	1.31 ± 0.07 ^c^	0.72 ± 0.05 ^a^
HDL-C (mmol/L)	2.86 ± 0.15 ^b^	2.20 ± 0.18 ^a^	2.71 ± 0.21 ^ab^
LDL-C/TC (%)	11.36 ± 0.63 ^a^	16.46 ± 1.46 ^b^	10.62 ± 1.10 ^a^
HDL-C/TC (%)	32.83 ± 2.12	27.67 ± 3.65	40.00 ± 4.47
TBA (μmol/L)	5.26 ± 0.14	6.03 ± 0.46	5.26 ± 0.20
TP (g/L)	22.78 ± 1.60	27.60 ± 1.73	24.63 ± 1.39

^abc^ Values with no or the same superscript letter mean no significant differences in the same row (*p* > 0.05), while different superscript letters indicate significant differences (*p* < 0.05).

**Table 6:** Effects of experimental diets on liver function of largemouth bass (Mean ± SEM, *n* = 8).

	HFM	HSB	HSB+P
AKP (U/g prot)	229.90 ± 24.34	181.00 ± 28.46	245.77 ± 30.86
AST (U/g prot)	21.62 ± 0.88	26.16 ± 3.91	24.79 ± 2.25
ALT(U/g prot)	136.15 ± 14.72	113.54 ± 21.47	136.88 ± 12.58
TBA (μmol/g prot)	2.96 ± 0.44 ^a^	5.96 ± 1.22 ^b^	2.68 ± 0.09 ^a^
TP (g prot/L)	62.92 ± 2.55	57.00 ± 2.95	57.99 ± 1.92

^ab^ Values with no or the same superscript letter mean no significant differences in the same row (*p* > 0.05), while different superscript letters indicate significant differences (*p* < 0.05).

**Table 7:** Effects of experimental diets on hepatic antioxidant capacity of largemouth bass (Mean ± SEM, *n* = 8).

	HFM	HSB	HSB+P
ROS (IU/mg prot)	35.17 ± 1.63 ^a^	42.79 ± 2.79 ^b^	34.87 ± 1.34 ^a^
T-AOC (mmol/mg prot)	106.89 ± 3.38 ^a^	127.06 ± 3.03 ^b^	105.55 ± 3.11 ^a^
T-SOD (U/mg prot)	888.46 ± 24.77	866.97 ± 21.56	848.30 ± 18.86
CAT (U/mg prot)	13.95 ± 0.88 ^a^	24.01 ± 1.06 ^b^	15.71 ± 1.25 ^a^
GSH-Px (U/mg prot)	35.32 ± 1.22 ^b^	24.43 ± 1.56 ^a^	41.14 ± 1.24 ^c^
MDA (nmol/mg prot)	0.76 ± 0.12	0.89 ± 0.12	0.80 ± 0.11

^abc^ Values with no or the same superscript letter mean no significant differences in the same row (*p* > 0.05), while different superscript letters indicate significant differences (*p* < 0.05).

## Data Availability

The data presented in this study are available on request from the corresponding author. The data are not publicly available due to containing information that could compromise the privacy of research participants.
